# Comparative analysis of clinical factors associated with pedicle screw pull‐out during or immediately after surgery between intraoperative cone‐beam computed tomography and postoperative computed tomography

**DOI:** 10.1186/s12891-020-03916-9

**Published:** 2021-01-09

**Authors:** Satoshi Sumiya, Kazuyuki Fukushima, Yoshiro Kurosa, Takashi Hirai, Hiroyuki Inose, Toshitaka Yoshii, Atsushi Okawa

**Affiliations:** 1 Department of Orthopaedic Surgery, Saku Central Hospital Advanced Care Center, 3400-28 Nakagomi, 385-0051 Saku City, Nagano Japan; 2 Department of Orthopaedic Surgery, Tokyo Medical and Dental University, Tokyo, Japan

**Keywords:** Pedicle screw pull‐out, Cone‐beam computed tomography (CBCT), Diffuse idiopathic skeletal hyperostosis (DISH), Connecting rod

## Abstract

**Background:**

No studies to date have elucidated the clinical factors associated with pedicle screw pull-out during or immediately after surgery. The aim of this study was to assess the frequency of pedicle screw pull-out by comparing intraoperative scans obtained using cone-beam computed tomography (CBCT) with postoperative scans obtained using computed tomography (CT). We also sought to determine the incidence of pedicle screw pull-out and identify relevant risk factors.

**Methods:**

This was a retrospective analysis of prospectively collected data for 742 pedicle screws placed in 76 consecutive patients who underwent at least triple-level posterior fixation for thoracic or lumbar spinal injury, spinal metastasis, or pyogenic spondylitis between April 2014 and July 2020. Pedicle screw pull-out distance in the axial and sagittal planes was compared between CT scans obtained 2 days postoperatively and CBCT images acquired intraoperatively. Risk factors associated with pedicle screw pull-out were investigated by multivariate logistic regression analysis.

**Results:**

Pedicle screw pull-out was seen with 58 pedicle screws (7.8%) in 26 patients (34.2%). There were significant differences in age, number of fused segments, frequency of diffuse idiopathic skeletal hyperostosis (DISH), and medical history of osteoporosis for pedicle screw pull-out. Risk factors for pedicle screw pull-out were older age (odds ratio 1.07, 95% confidence interval 1.02–1.130) and a diagnosis of DISH (odds ratio 3.35, 95% confidence interval 1.12–10.00). Several cases suggest that use of connecting rods was an important factor in intraoperative pedicle screw pull-out.

**Conclusions:**

Our findings suggest that age, number of fused segments, presence of DISH, and medical history of osteoporosis are risk factors for pedicle screw pull-out, with the greatest being older age and DISH.

## Text

### Background

Many authors have described the efficacy of posterior pedicle screw instrumentation [1, 2], which is widely used in patients with spinal diseases. In recent years, the use of long instrumented fusion with pedicle screw has increased in patients with trauma, degenerative diseases, and deformity. However, pedicle screw misplacement may cause not only serious complications such as neurovascular injury [3, 4], but also delayed complications resulted by inadequate strength and result to delayed complications such as screw loosening and nonunion. In addition the underlying osteoporosis can caused screw loosening due to in adequate strength at the screw bone interface. The inadequate strength of the implant construct lead to the correction loss or nonunion and may result to neurological impairment, back pain or neuropathy [4, 5].

Nowadays, many institutions can perform spinal instrumentation surgery using intraoperative cone-beam computed tomography (CBCT) in a hybrid operating room [6, 7] or an O-arm imaging system (Medtronic, Minneapolis, MN) [8, 9]. CBCT is a three-dimensional (3D) imaging modality that reconstructs projection data obtained by a rotational C-arm with a flat panel detector [7]. It can visualize low-contrast objects, such as soft tissue or small vessels, as well as high-contrast structures, including enhanced vessels or bone. Several authors have reported the utility of CBCT [6, 7], and thus it is being increasingly used in spine surgeries. The recent advent of a 3D-CT-based navigation system for intraoperative CBCT has improved the accuracy of pedicle screw insertion. Further, intraoperative CBCT has made it possible to confirm the presence or absence of screw deviation during surgery.

Long instrumented fusion requires insertion of a large number of pedicle screws, and the above-mentioned intraoperative modalities help to ensure surgical safety. The position of the pedicle screws can be confirmed using intraoperative CBCT in a hybrid operating room after pedicle screw insertion before rod connection. We have encountered several cases of intraoperative pedicle screw pull-out as a result of rod connection during posterior thoracic or lumbar spine surgery. In long instrumented fusion in particular, pedicle screw pull-out was found to be a risk factor for pedicle screw loosening [10]. It has been reported that pedicle screw pull-out can occur during, but not after, surgery [10, 11]. A previous study investigated percutaneous pedicle screw (PPS) pull-out during rod reduction and reported its association with screw loosening. However, the clinical factors associated with pedicle screw pull-out have not been elucidated [10].

In this study, we investigated the frequency of pedicle screw pull-out by comparing intraoperative CBCT images and postoperative CT images acquired 2 days after surgery. We also sought to identify and evaluate the relevant risk factors for screw pull-out.

## Materials and methods

This study was approved by the Institutional Ethics Committee (Approval No. R201904-06). In this study, we conducted a retrospective analysis of prospectively collected data from 76 consecutive patients who underwent long posterior spinal instrumented fixation at 3 or more levels for thoracic or lumbar spinal injury, spinal metastasis, or pyogenic spondylitis between April 2014 and July 2020. The patients were evaluated using CBCT intraoperatively in a hybrid operating room. Evaluation by CT was performed again on postoperative day 2 before mobilization. Demographic and surgical data collected included age, sex, body mass index (BMI), operating time, estimated blood loss, underlying disease, number of fused segments, use of a Hook system, PPS insertion, presence of diffuse idiopathic skeletal hyperostosis (DISH), medical history of osteoporosis, smoking, preoperative comorbidities, degree of sagittal alignment, alignment change in the sagittal plane, screw density, type of rod, and screw design.

### Surgical procedure

**Fig. 1:**
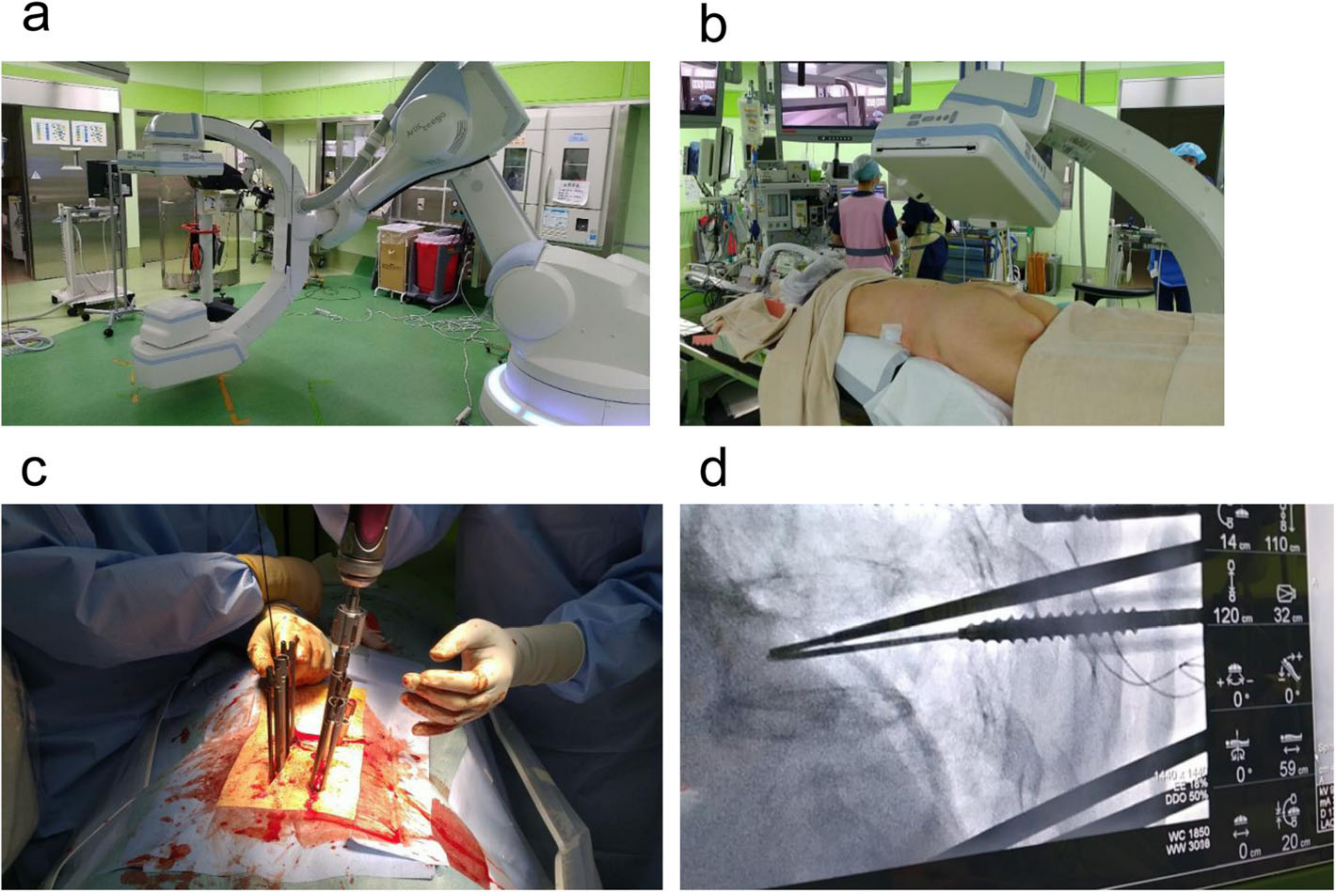
Photograph showing the hybrid operating room. **a** The Artis Zeego system, a 3D imaging tool that reconstructs projection data obtained by a rotational C-arm with a flat panel detector. **b** The patient is placed prone with the trunk on a radiolucent operating table. **c** Percutaneous pedicle screw insertion. **d** The targeting needle is inserted into the pedicle, which is projected onto the Zeego control screen

All patients underwent posterior spinal fixation in the prone position with the trunk on a radiolucent operating table in a hybrid operating room. Following anesthesia, standard surgical exposure was performed via a midline skin incision using an open approach. PPS placement was performed via a 1.5-cm stab incision made laterally. The targeting needle was inserted into the pedicle at the superolateral border based on the anterior-posterior view under rotational C-arm fluoroscopic guidance. The guidewire was inserted through the targeting device and into the pedicle. The pedicle was then tapped using the guidewire. Next, the pedicle screw was inserted over the guidewire. CBCT images were obtained at this timing to confirm the screw position. Finally, rods were installed aligned to each screw placed in the open surgery and were inserted starting from the most cranial skin incision in the PPS surgery. A new automatic rod bending system called the Bendini system (NuVasive, San Diego, USA) was not use in this study. The order in which the set-screws were installed varied among the operators (Fig. 1). In all surgeries, the rod was attached without correction and dekyphosis was performed. The same surgical method was used to operate on the thoracic and lumbar spine. A Solera Voyager spinal system (Medtronic, Memphis, TN) or Reline spinal system (NuVasive, San Diego, CA) was used for posterior spinal fixation. Mixed-thread and dual-thread screw designs were used. The types of rods were titanium and cobalt chrome. The operations were performed by 5 spine surgeons.

### Evaluation of screw position

In all cases, the implant position was assessed intraoperatively using CBCT scans obtained using a DynaCT system (Artis Zeego; Siemens Healthcare, Erlangen, Germany), a 3D imaging tool that reconstructs projection data obtained by a rotational C-arm with a flat panel detector. The series consisted of 0.616-mm CBCT sections reconstructed at 0.616-mm intervals. Raw data were used to reconstruct axial 2.0-mm-thick CBCT sections every 2.0 mm with a field of view adequate for visualization of the spine, as well as sagittal and coronal reformatted images of the thoracolumbar spine. The position of the implant was assessed postoperatively on CT scans obtained using a 320-rowarea-detector CT system (Toshiba Medical Systems, Tokyo, Japan). The series consisted of 0.5-mm-thick CT sections reconstructed at 0.5-mm intervals. Raw data were used to reconstruct 2.0-mm-thick axial CT sections every 2.0 mm with a field of view that was adequate for spine visualization and for sagittal and coronal reconstruction of the thoracolumbar spine.

**Fig. 2:**
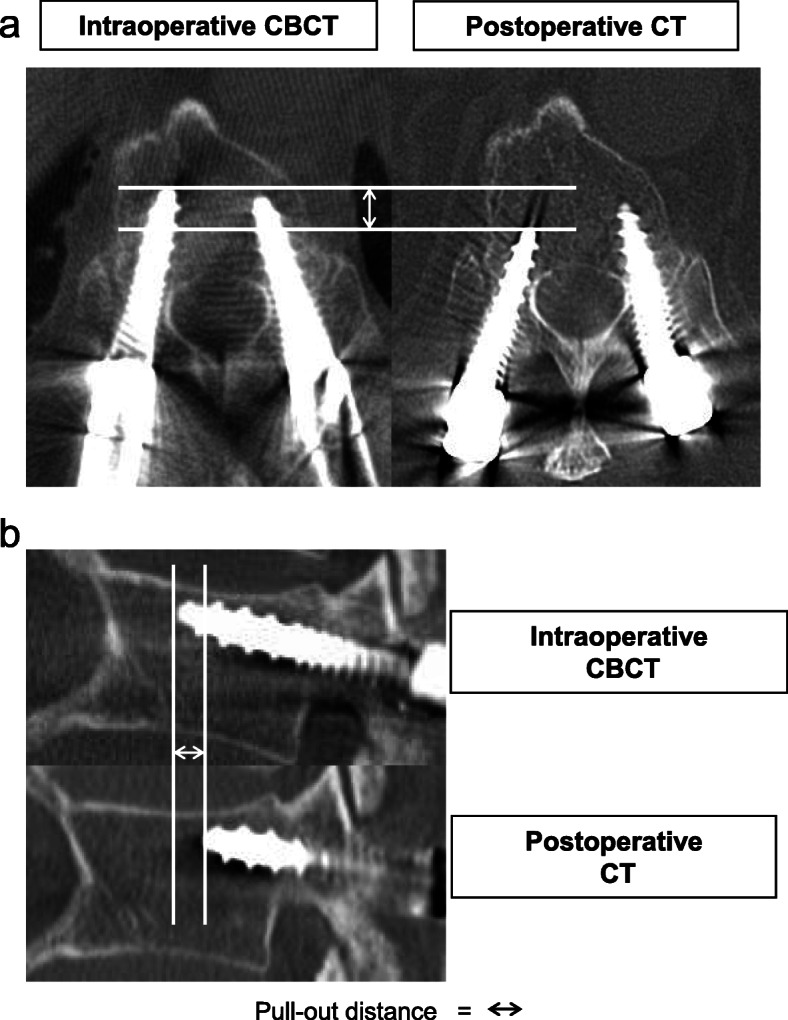
Evaluation of pedicle screw pullout distance. Pedicle screw pull-out was defined as a distance of > 2 mm in the (**a**) axial and (**b**) sagittal CBCT and CT planes. CBCT, cone-beam computed tomography; CT, computed tomography

Screw misplacement was classified according to the system devised by Schizas et al. [12]. Screw malposition was categorized as minor (< 3 mm), moderate (3–6 mm), or severe (> 6 mm) and the direction of perforation was classified as medial or lateral. Screw pull-out distances measured in the axial and sagittal planes on CT scans obtained 2 days postoperatively were compared with those on intraoperative CBCT images by two independent observers. Screw pull-out was defined as a change of more than 2 mm in the axial and sagittal views on postoperative CT compared with the position on intraoperative CBCT (Fig. 2). Data were also compared between the cases with screw pull-out and cases without screw pull-out. Risk factors for pedicle screw pull-out were identified by multivariate analysis.

**Fig. 3:**
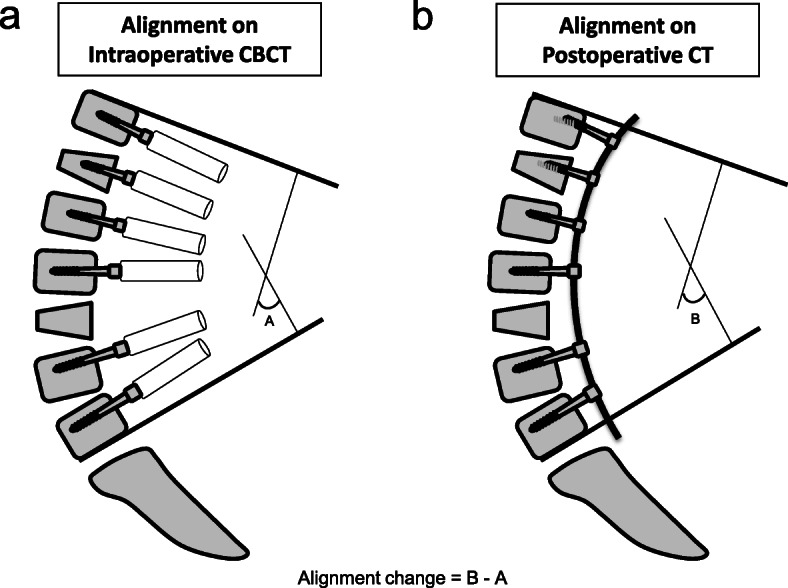
Evaluation of alignment. **a** Degree of sagittal alignment on intraoperative CBCT. **b** Degree of sagittal alignment in postoperative CT. Alignment change in the sagittal plane is the difference between (**a**) and (**b**)

Other factors assessed were the degree of sagittal alignment between the upper instrumented vertebra (UIV) and lower instrumented vertebra (LIV) on intraoperative CBCT or postoperative CT, alignment change in the sagittal plane on intraoperative CBCT to postoperative CT (Fig. 3), and screw density. Screw density was defined as the number of fixation screws divided by the number of available anchor sites from the UIV to the LIV.

### Statistical analysis

Statistical analysis was performed using Fisher’s exact test and the Mann-Whitney*U* test, with statistical significance set at P < 0.05. Risk factors were determined using logistic regression analysis with a forward stepwise procedure (P < 0.1 for entry). The threshold of alpha and forward selection was chosen to stabilize the statistical model during stepwise covariate selection considering the relatively small number of our outcomes. Inter-observer agreement was measured using kappa coefficient scores. Any discrepancy between the observers was resolved by discussion. All statistical analysis was performed using JMP version 13.1.0 software (SAS Institute Inc., Cary, NC).

## Results

A total of 742 pedicle screws were inserted in the thoracic or lumbar spine of the 76 patients. Patient demographics are shown in Table 1. Mean age of the 47 men and 29 women was 69.2 ± 14.4 years (range, 25–93 years). The diagnosis was spinal injury in 55 cases (72.4%), spinal metastasis in 15 (19.7%), and pyogenic spondylitis in 6 (7.9%). Mean number of fused segments was 4.8 ± 1.2. A hook system was used in 6 cases (7.9%) and the PPS method was used in 57 cases (75.0%). Twelve patients (15.8%) were smokers. Other diagnoses included DISH (*n* = 28, 36.8%), medical history of osteoporosis (*n* = 33, 43.4%), rheumatoid arthritis (*n* = 2, 2.6%), diabetes (*n* = 16, 21.1%), and asthma (*n* = 6, 7.9%). Three patients (3.9%) were on dialysis. A titanium rod was used in 60 cases (78.9%) and a cobalt chrome rod was used in 16 cases (21.1%). Mixed-thread screws were used in 15 cases (19.7%) and dual-thread screw were used in 61 cases (80.3%). Mean kyphotic angle of sagittal alignment between UIV and LIV was 6.2 ± 23.5 deg on intraoperative CBCT and 5.8 ± 23.5 deg on postoperative CT. Mean alignment change in the sagittal plane from intraoperative CBCT to postoperative CT was 2.1 ± 1.5 deg. Mean screw density was 1.69 ± 0.23 (screws/level).

**Table 1:** Patient demographics and clinical details

Variable	*N* = 76
Patients with pedicle screw pullout	26 (34.2)
Age, years	69.2 ± 14.4
Sex	
Male	47 (61.8)
Female	29 (38.2)
Body mass index	22.0 ± 3.4
Operating time, min	174.3 ± 85.7
Estimated blood loss, mL	225.3 ± 245.8
Disease	
Injury	55 (72.4)
Metastasis	15 (19.7)
Spondylitis	6 (7.9)
Segments fused, n	4.8 ± 1.2
Surgical procedure	
PPS	57 (75.0)
Open	19 (25.0)
Use of a hook system	6 (7.9)
Preoperative complications	
DISH	28 (36.8)
Osteoporosis	33 (43.4)
Rheumatoid arthritis	2 (2.6)
Diabetes	16 (21.1)
Asthma	6 (7.9)
Requirement for dialysis	3 (3.9)
Smoking	12 (15.8)
Type of rod	
Titanium	60 (78.9)
Cobalt chrome	16 (21.1)
Screw designs	
Mixed thread	15 (19.7)
Dual thread	61 (80.3)
Degree of sagittal alignment between the UIV and LIV (kyphotic angle)	
Intraoperative CBCT, deg	6.2 ± 23.5
Postoperative CT, deg	5.8 ± 23.5
Alignment change in the sagittal plane from CBCT to CT, deg	2.1 ± 1.5
Screw density, screws/level	1.69 ± 0.23

Data are presented as mean ± standard deviation or as number (%). *CBCT* cone-beam computed tomography, *CT* computed tomography, *DISH* diffuse idiopathic skeletal hyperostosis, *LIV* lower instrumented vertebra, *PPS* percutaneous pedicle screw, *UIV* upper instrumented vertebra

**Fig. 4:**
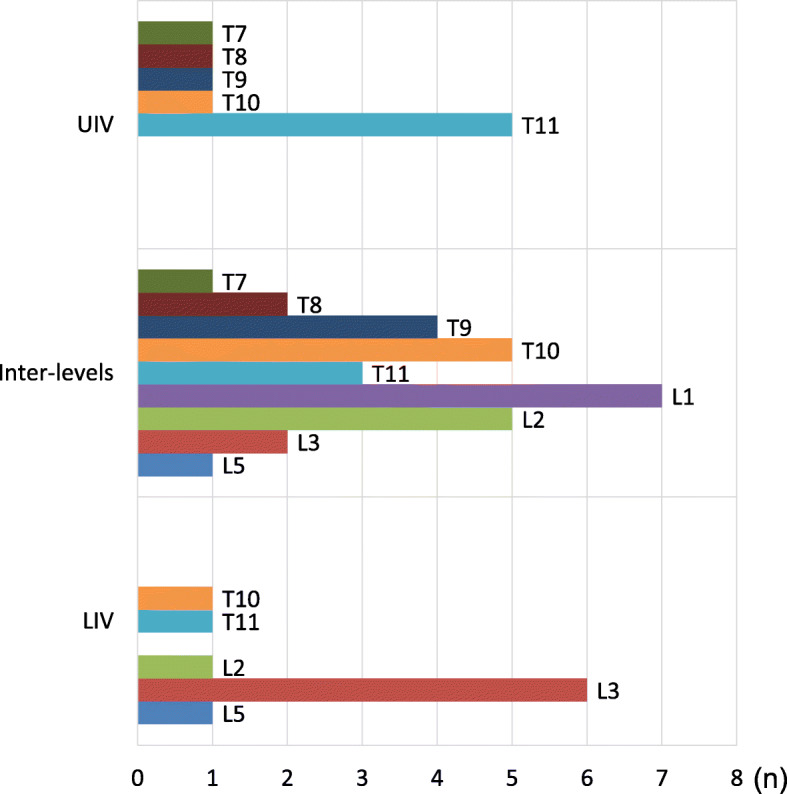
Distribution and frequency of pedicle screw pull-out. UIV, upper instrumented vertebra; LIV, lower instrumented vertebra

Screw pull-out occurred for 58 of the 742 pedicle screws (7.8%) inserted, in 26 of the 76 patients (34.2%). For almost all cases of pull-out, insertion had been via PPS. The pedicle screw-related variables are shown in Table 2. Twenty-eight of the pedicle screws (3.8%) were found to be misplaced on postoperative CT views. Sub-classificationanalysis revealed minor perforation of 14 screws (1.9%), moderate perforation of 8 screws (1.1%), and severe perforation of 6 screws (0.8%). Both pull-out and misplacement occurred with 7 screws (0.9%). Pull-out screws were observed in the upper instrumented vertebra in 9 cases (15.5%), in the lower instrumented vertebra in 11 (19.0%), and in the inter-levels in 38 (65.5%; Fig. 4). There was substantial inter-observer agreement in judging screw pull-out distance (κ = 0.70). All values indicated substantial agreement.

**Table 2:** Pedicle screw-related variables

Variable	*N* = 742
Adequate insertion	714 (96.2)
Misplacement	28 (3.8)
Penetration	
Minor	14 (1.9)
Moderate	8 (1.1)
Severe	6 (0.8)
Pedicle screw pull-out	58 (7.8)
Pedicle screw pull-out and misplacement	7 (0.9)
Pull-out area	
Upper instrumented vertebra	9 (15.5)
Inter-levels	38 (65.5)
Lower instrumented vertebra	11 (19.0)

Data are presented as number (%)

There was no significant difference in sex, BMI, operating time, estimated blood loss, underlying disease, use of a Hook system, surgical procedure, smoking status, preoperative comorbidities, degree of sagittal alignment, alignment change in the sagittal plane, screw density, type of rod, or screw design between the non-pull-out and pull-out groups. Patients in the pull-out group were more likely to be elderly, have more fused segments, have a diagnosis of DISH, and have a medical history of osteoporosis (Table 3).

**Table 3:** Comparison of the screw pull-out group and the normal group

Variable	Non-pull-out group	Pull-out group	***P***-value
Patients	50 (65.8%)	26 (34.2%)	
Age, years	65.2 ±13.7	76.9 ± 12.5	*P* = 0.0001*
Sex			
Male	33	14	
Female	17	12	*P* = 0.33
Body mass index	22.1 ± 3.7	21.8 ± 2.8	*P* = 0.80
Operating time, min	161.3 ± 76.8	199.3 ± 97.4	*P* = 0.10
Estimated blood loss, g	229.1 ± 269.4	217.9 ± 197.1	*P* = 0.87
Disease			
Injury	35	20	*P* = 0.60
Metastasis	11	4	*P* = 0.56
Spondylitis	4	2	*P* = 1.00
Fused segments, n	4.6 ± 1.3	5.3 ± 1.0	*P* = 0.0031*
Surgical procedure			
PPS	38	19	
Open	12	7	*P* = 0.79
Use of a hook system	2	4	*P* = 0.17
Preoperative complications			
DISH	12	16	*P* = 0.0023*
Osteoporosis	15	17	*P* = 0.0038*
Rheumatoid arthritis	1	1	*P* = 1.00
Diabetes	9	7	*P* = 0.39
Asthma	6	0	*P* = 1.00
Requirement for dialysis	2	1	*P* = 1.00
Smoking	11	1	*P* = 1.00
Type of rods			
Titanium	39	21	
Cobalt chrome	11	5	*P* = 1.00
Screw designs			
Mixed thread	11	4	
Dual thread	39	22	*P* = 1.00
Degree of sagittal alignment between the UIV and LIV (kyphotic angle)			
Intraoperative CBCT, deg	6.5 ± 24.3	5.3 ± 22.3	*P* = 0.81
Postoperative CT, deg	5.9 ± 24.3	5.7 ± 22.5	*P* = 0.97
Alignment change in the sagittal plane from CBCT to CT, deg	2.2 ± 1.6	1.9 ± 1.3	*P* = 0.55
Screw density, screws/level	1.69 ± 0.24	1.70 ± 0.21	*P* = 0.41

Data are presented as mean standard deviation or as number unless otherwise stated. *CBCT* cone-beam computed tomography, *CT* computed tomography, *DISH* diffuse idiopathic skeletal hyperostosis, *LIV* lower instrumented vertebra, *PPS* percutaneous pedicle screw, *UIV* upper instrumented vertebra. *P < 0.05

The risk factors for pedicle screw pull-out were evaluated using logistic regression analysis. From univariate analysis, the dependent variable was defined as the presence of screw pull-out and the independent variables were age, number of fused segments, DISH, medical history of osteoporosis, and PPS procedure. As a result, the independent risk factors identified were older age (odds ratio 1.07, 95% confidence interval 1.02–1.130, P = 0.0098) and DISH (odds ratio 3.35, 95% confidence interval 1.12–10.00, P = 0.0302; Table 4).

**Table 4:** Multivariate logistic regression analysis of risk factors for pedicle screw pull-out

Risk factor	Odds ratio	95% CI	***P***-value
Age	1.07	(1.02–1.130)	*P* = 0.0098
DISH	3.35	(1.12–10.00)	*P* = 0.0302

*CI* confidence interval, *DISH* diffuse idiopathic skeletal hyperostosis

## Discussion

Previous reports have shown that pedicle screw loosening after surgery is a serious complication of spinal fixation surgery. Screw loosening causes nonunion back pain and sometimes neurological impairment, and it can be an indication for reoperation [13, 14]. Ohba et al. reported that pedicle screw pull-out was a risk factor for postoperative screw loosening [10]. In the present study, we investigated the incidence of screw pull-out and attempted to identify relevant risk factors.

In our study, pull-out occurred for a total of 58 pedicle screws in 26 cases, giving an overall pedicle screw pull-out rate of 7.8%, which is relatively low compared with the rate of 16.2% reported previously [10]. Although screw pull-out has been defined as a distance of ≥ 1 mm in previous studies, we defined it as ≥ 2 mm in our study. The difference in the cutoff value may explain why the incidence of screw pull-out was lower in this study than in previous investigations. Pedicle screw pull-out was detected in 34.2% of the patients in this study. Of note, all the postoperative CT images were obtained on postoperative day 2 before the patients started to mobilize, meaning that screw pull-out is most likely to occur during the operation. Other studies also mentioned that pedicle screw pull-out occurs during rod connection [10, 11]. Generally, screw pull-out can occur if there is a gap between the shape of the rod and the actual spinal alignment. In the PPS system especially, pull-out may easily occur at the time of inserting set screws because the gap between the rod and the screw head is not visible [10, 11]. Furthermore, we found that pedicle screw pull-out could occur at any of the levels including the cranial end, caudal end and inter-levels. The force in the direction in which the screw comes off is considered to vary according to the order in which the set screws are placed.

In this study, patients in the pull-out group were more likely to be elderly, to have more fused segments, and to have a diagnosis of DISH or a medical history of osteoporosis. Other researchers have also identified older age and osteoporosis as risk factors for pedicle screw loosening [13, 15]. The number of fused segments was also associated with screw pull-out. In long fusion, force may be applied to the direction in which the rod does not fit the screw head and the force on the lever arm of the rod is increased, thereby increasing the risk of pedicle screw pull-out compared with short fusion. Additionally, in long spinal fusion, there are multiple screws to be connected to the rod, making it difficult to achieve appropriate rod-bending and to fit the rod completely to each screw head.

Logistic regression analysis identified advanced presence of DISH to be independent risk factors for pedicle screw pull-out. DISH appears on radiographs and CT images as ossification along the anterolateral aspect of the vertebral bodies [16, 17]. Therefore, movement of the spine becomes limited by spinal ossification. Given that DISH restricts the segmental motion of the spine, the screw-rod system applies force in the direction in which the screw comes off if the fused segments are over-corrected by de-kyphotic rod placement. Additionally, although there was no significant difference in DISH in this study, DISH is often treated with PPS surgery, and it is considered that the screw comes off because the fitting between the rod and the screw head cannot be checked directly. Patients with DISH also tend to be elderly and have poor bone quality [18–20]. Therefore, posterior segmental fusion extending at least three levels above and below has been recommended in patients with DISH [21]. The significantly higher number of fused segments in the pull-out group was considered to be because patients with DISH often require long fusion. To our knowledge, there have been no reports showing that the presence of DISH is associated with pedicle screw pull-out, and ours is the first to clearly demonstrate that DISH is a significant risk factor for intraoperative screw pull-out with an odds ratio of 3.35. Although a medical history of osteoporosis has not been previous identified as a risk factor for screw pull-out, osteoporosis is considered an important factor in screw pull-out [13, 15]. Because this study included many patients who underwent emergency surgery, there are many cases where bone density could not be measured preoperatively. If bone density had been measured before surgery, there might have been more patients with osteoporosis, which would result in a higher rate of screw pull-out.

The most important factor is to ensure appropriate rod bending and to gently connect the rod to each screw head. As it is sometimes difficult to create a perfect curve by manual bending, a new automatic rod bending system may be useful to improve the fitting of the rod [22]. Also, a new pedicle screw device using cement augmentation has recently become available as a strategy to prevent pull-out [23].

There are several limitations in this study. First, we did not evaluate the screw length, diameter, position, or trajectory as factors affecting pull-out. Second, radiation exposure is a risk for patients, although we used intraoperative CBCT, in which the radiation exposure is reduced. Third, other important factors in preventing screw pull-out in the setting of in situ fusion are how perfectly the rod is contoured to the screws and how perfectly the head of each screw is aligned in the same plane. These technical issues could not be assessed with the present study design. Fourth, the sample size was limited; Nevertheless, this study identified age and presence of DISH as important risk factors for screw pull-out during surgery.

## Conclusions

This study analyzed the clinical factors associated with pedicle screw pull-out by comparing findings on both intraoperative CBCT and postoperative CT. Our findings suggest that age, number of fused segments, presence of DISH, and medical history of osteoporosis are risk factors for pedicle screw pull-out, with the greatest being older age and DISH.

## Data Availability

The data utilized are accessible from the corresponding author upon reasonable request.

## Abbreviations

BMI: Body mass index
CBCT: Cone-beam computed tomography
CT: Computed tomography
DISH: Diffuse idiopathic skeletal hyperostosis
LIV: Lower instrumented vertebra
PPS: Percutaneous pedicle screw
UIV: Upper instrumented vertebra
